# Motivators and barriers to COVID-19 vaccination of healthcare workers in Latvia

**DOI:** 10.3389/fpsyg.2022.903506

**Published:** 2022-10-05

**Authors:** Sintija Lielsvagere-Endele, Jelena Kolesnikova, Elina Puzanova, Svetlana Timofejeva, Inga Millere

**Affiliations:** ^1^Psychology Laboratory, Faculty of Public Health and Social Welfare, Riga Stradins University, Riga, Latvia; ^2^Faculty of Public Health and Social Welfare, Riga Stradins University, Riga, Latvia

**Keywords:** COVID-19 vaccination, healthcare workers, vaccine hesitancy, vaccine attitudes, vaccination motivators, vaccination barriers

## Abstract

This study aims to identify motivators and barriers regarding Coronavirus disease 2019 (COVID-19) vaccination among Latvian healthcare workers (HCWs). Data were collected from March to May 2021 using an online survey. Overall, 1,444 participants took part in the study. From this pool of respondents, 528 indicated motivating factors in favor of the COVID-19 vaccination (86.5% were women; aged between 20 and 75 years), while 198 mentioned barriers against the COVID-19 vaccination (92.9% were women; aged between 19 and 68 years). The thematic analysis was conducted on two open-ended questions. The main motivators reported for COVID-19 vaccination were belief in the effectiveness of the vaccine, benefits of easing COVID-19 restrictions, responsibility, and restriction or pressure in case of non-vaccination. The main barriers reported regarding the COVID-19 vaccination were concerns about the effectiveness and safety of vaccines, perceived health risks of vaccination, risk perception toward COVID-19, misinformation about COVID-19 vaccines, belief that vaccination is being imposed, and belief in the conspiracy theories surrounding COVID-19. The results of this study help identify the existing motivating and hindering factors for COVID-19 vaccination among HCWs in Latvia. These results can be used to promote vaccination in HCW, develop information campaigns, and alleviate concerns of HCW.

## Introduction

The novel coronavirus SARS-CoV-2, which causes Coronavirus disease 2019 (COVID-19), was first detected in Wuhan, China, in late 2019 and rapidly spread across the world. The World Health Organization (2020) declared the COVID-19 outbreak a pandemic in March 2020. The first case of the disease in Latvia was detected on 2 March 2020 (The Ministry of Health of the Republic of Latvia, 2020).

Apart from preventive measures imposed by governments around the world to limit the transmission of the virus, vaccination presented itself as the most efficient and least financially harmful solution. On 21 December 2020, the first COVID-19 vaccine (“Comirnaty,” developed by BioNTech and Pfizer) was already approved and authorized for use across the European Union by the European Medicines Agency (2020). On 28 December 2020, Latvia started providing COVID-19 vaccines for the first priority group–healthcare workers (HCWs), who were directly exposed to COVID-19 patients daily. After the first priority group, the possibility to receive the vaccine was extended to other groups of residents (Latvian Centre for Disease Prevention and Control, 2020).

The success of every immunization campaign depends on its reach, but during the COVID-19 pandemic, vaccination hesitancy (Sallam, 2021), misinformation (Garrett, 2020), as well as protests against any coronavirus-related restrictions and vaccination became a global problem. The HCWs were expected to educate residents on vaccine-related questions and support the immunization process. However, a significant proportion of them was not willing to be vaccinated themselves (Hajure et al., 2021) and might have also discouraged patients from taking this step (European Centre for Disease Prevention and Control, 2015).

Several studies have shown that HCW acceptance of COVID-19 vaccination varies across countries from 27.7% in the Democratic Republic of the Congo (Nzaji et al., 2020) to 96.2% in several Asian countries (Chew et al., 2021). A survey conducted by the research center, SKDS in January 2021, shows that 32% of Latvian residents were ready to be vaccinated at the first opportunity, whilst 40% wanted to wait and assess the situation in more detail (The Cabinet of Ministers of the Republic of Latvia, 2021). HCWs in Latvia (*N* = 1,444) have shown accepting attitudes toward the COVID-19 vaccination in 76.1% of cases (Lielšvāgere-Endele et al., 2021).

In 2012, the SAGE group working on vaccine hesitancy defined vaccine hesitancy as the: “[…] delay in acceptance or refusal of vaccines despite availability of vaccination services. Vaccine hesitancy is complex and context-specific, varying across time, place, and vaccine types. It is influenced by factors such as complacency, convenience, and confidence.” One of the factors contributing to vaccine hesitancy is the introduction of novel vaccines (World Health Organization, 2014). There are different hindering and motivating factors for receiving the vaccine. Currently, researchers have found that barriers to COVID-19 vaccination among HCWs could be related to concerns about health safety, vaccine efficacy, and the vaccine development process (Dzieciolowska et al., 2021; Gagneux-Brunon et al., 2021; Verger et al., 2021), as well as fear (Papagiannis et al., 2021), misinformation (Nzaji et al., 2020; Dzieciolowska et al., 2021), and insufficient time for decision-making (Hajure et al., 2021). On the other hand, high self-perceived risk, perceived impact of the virus on health, high risk of contracting COVID-19 at the workplace (Hajure et al., 2021; Li et al., 2021), as well as chronic illnesses, and willingness to act preventively and trust in government (Hajure et al., 2021) are the main motivators to get vaccinated among HCWs. The main themes related to COVID-19 vaccination among HCWs are negative emotions, the vaccine and vaccination process, social media influence, political processes, attitude, intention to be vaccinated, and trust. These themes are related both to an accepting attitude and hesitancy toward vaccination (Aci et al., 2021).

Despite vaccine hesitancy, multiple countries have already made COVID-19 vaccination mandatory for HCWs (Sokol, 2021). As a result, a portion of specialists had to leave their hospital jobs, whilst some reluctantly agreed to be vaccinated. It should be noted that there is also a large percentage of HCWs who accept COVID-19 vaccines and motivate others to get vaccinated. Arguments for and against COVID-19 vaccination among HCWs still require further research. Therefore, this study aims to explore motivators and barriers to COVID-19 vaccination of HCWs in Latvia.

## Materials and methods

### Research design

Data were obtained in the cross-sectional survey study, “Latvian healthcare workers’ attitude toward COVID-19 vaccination” and included both closed- and open-ended questions. In this study, two open-ended questions from the survey were analyzed, using the qualitative data analysis method of thematic analysis.

### Sample

Respondents came from nine hospitals located in different towns in Latvia during the timeframe of March to May 2021. The survey included both closed- and open-ended questions about HCW motivating (motivators) and hindering (barriers) factors regarding the COVID-19 vaccination. In total, 1,444 respondents took part in the survey, but only those who answered the open-ended questions are included in this analysis. The answers to the open-ended questions were not mandatory. Therefore, the number of respondents to motivating and hindering factors varies. Each respondent had the option of indicating both motivating factors and barriers or not indicating anything at all. Respondents who gave unspecified answers such as “I do not want to” or “I have already been vaccinated,” etc., as well as those who work in a hospital but are not HCWs (administration and technical staff), were excluded from the analysis. As a result, the analysis included 198 responses about respondents’ vaccination barriers and 528 responses for vaccination motivating factors.

In total, 528 respondents (86.5% of them women) indicated motivating factors in favor of the COVID-19 vaccination. The age of respondents in this group ranged from 20 to 75 years (*M* = 41.81; SD = 13.25). Respondents exercised different professions, most of them were doctors (28.22%) and nurses (29.17%). In addition, most respondents had at least a bachelor’s degree (73.87%) (see Table 1).

**TABLE 1:** Sociodemographic characteristics of healthcare worker (HCW).

	Mentioned barriers (*n* = 198)	Mentioned motivators (*n* = 528)
Age	19–68 (*M* = 39.66; SD = 12.42)	20–75 (*M* = 41.81; SD = 13.25)
**Sex**		
Women	184 (92.93%)	457 (86.55%)
Men	14 (7.07%)	71 (10.42%)
**Professions**		
Doctor	18 (9.09%)	149 (28.22%)
Resident	8 (4.04%)	47 (8.90%)
Nurse	84 (42.42%)	154 (29.17%)
Medical assistant	5 (2.53%)	14 (2.65%)
Midwife	3 (1.52%)	15 (2.84%)
Physician’s assistant	6 (3.03%)	14 (2.65%)
Assistant nurse	29 (14.65%)	30 (5.68%)
Junior nurse assistant	20 (10.10%)	33 (6.25%)
(sanitary)		
Other specialists	25 (12.63%)	72 (13.64%)
**Education level**		
Basic education	3 (1.52%)	6 (1.14%)
Vocational education	1 (0.51%)	1 (0.19%)
General secondary education	9 (4.55%)	14 (2.65%)
Vocational secondary	35 (17.68%)	49 (9.28%)
education		
Incomplete higher education	20 (10.10%)	31 (5.87%)
or studying		
College education	25 (12.63%)	37 (7.01%)
Bachelor’s degree	62 (31.31%)	140 (26.52%)
Master’s degree or Doctor of	39 (19.70%)	228 (43.18%)
Medicine degree		
Doctorate	4 (2.02%)	22 (4.17%)

In total, 198 respondents (92.9% of them women) described barriers against COVID-19 vaccination. The age of respondents in this group ranged from 19 to 68 years (*M* = 39.66; SD = 12.42). Respondents exercised different professions, most of them were nurses (42.42%). In addition, most respondents had at least a bachelor’s degree (53.03%) (see Table 1).

### Data collections

This study used part of the survey “Healthcare workers’ attitude toward COVID-19 vaccination” (Lielšvāgere-Endele et al., 2021). The survey has a total of 59 questions, including demographic characteristics. A total of 54 questions were closed, and five were open. The survey was first approved by hospital management and then sent out to hospital staff by the hospital management representative through e-mail, an internal information system, and/or short message service (SMS). The survey was conducted online, anonymously, and voluntarily. Informed consent was obtained from all subjects involved in the study. The demographic characteristics (age, sex, education, and profession) and respondents’ answers to two open-ended questions (“What are your arguments for not being vaccinated?” and “What are your arguments for being vaccinated?”) were analyzed in this study.

### Data analysis

Braun and Clarke’s (2019) six-phase approach to reflexive thematic analysis (Clarke et al., 2012) was used for analyzing data of respondents’ responses to two open-ended questions. It is the most delineated method of conducting thematic analysis (Byrne, 2022). The survey responses were assessed through data familiarization, the generation of initial codes, generating themes, reviewing potential themes, defining and naming themes, and producing the report. Two independent researchers coded data manually to reduce the risk of observer bias.

Additionally, the researchers quantified the themes (by percentage) to determine the frequency of responses to specific themes. It allowed the study to highlight the most common topical themes among HCWs during vaccination in the hospitals of Latvia.

## Results

Using thematic analysis, we identified 6 themes of vaccination barriers which are presented in Section “Coronavirus disease 2019 vaccination barriers.” We also identified 4 themes of vaccination motivators which are presented in Section “Coronavirus disease 2019 vaccination motivator.” Each of the themes covers several sub-themes that were mentioned by the respondents in their responses. To provide an example, the responses of some respondents are also presented for each topic. The frequency with which themes occurred is stated in Table 2 for vaccination barriers and in Table 3 for vaccination motivators.

**TABLE 2:** Coronavirus disease 2019 (COVID-19) vaccination barriers (*n* = 198).

Qualitative themes	Percentage (%)
Concerns about the effectiveness and safety of vaccines	41.92%
Perceived health risk of vaccination	41.91%
Risk perception toward COVID-19	36.87%
Misinformation about COVID-19 vaccines	9.60%
Belief that vaccination is being imposed	5.56%
Belief in conspiracy theories surrounding COVID-19	2.53%

Participants could refer to more than one barrier.

**TABLE 3:** Coronavirus disease 2019 (COVID-19) vaccination motivators (*n* = 528).

Qualitative themes	Percentage (%)
Belief in the effectiveness of the vaccine	88.26%
Benefits of easing COVID-19 restrictions	19.89%
Responsibility	15.53%
Restriction or pressure in case of non-vaccination	5.30%

Participants could refer to more than one motivator.

### Coronavirus disease 2019 vaccination barriers

#### Theme: Concerns about the effectiveness and safety of vaccines

Among HCWs, the most important argument for not vaccinating is their doubts about whether the vaccine is effective and safe. Perhaps, this also has a great influence on the formation of the belief that vaccines can harm their health.

“*I would get vaccinated if it would 100% rule out the risk of getting COVID-19, but if I can still get it anyway (only in a milder form), I don’t see the point in doing so*” (#1,243)

Some of the workers have expressed the opinion that the COVID-19 vaccination will need to be repeated, therefore they are not ready to be vaccinated now.

“*Not sure about the effectiveness of vaccines. If you have to get vaccinated every year, it’s better later*” (#517)

There were expressed distrust and concerns about the speed of the vaccine production. One of the respondents expressed his dissatisfaction by saying that he would not become a test subject.

“*Inadequately created, within a few months*” (#496);

“*The vaccine has not been studied. TEST BUNNIES*” (#273)

Although HCWs could be the first to be vaccinated, the reasons for delaying it for some of them were the unavailability of all known vaccines at that time and the belief that vaccines were not well understood. Some of them stated that they opposed certain vaccines or that the preferred vaccine was unavailable.

“*I am waiting for a vaccine (Jonson & Jonson) that has been studied more on the most contagious types of COVID (UK, South Africa)*” (#264)

#### Theme: Perceived health risk of vaccination

The following important argument against the vaccination of HCWs is their concern about the potential adverse risk of vaccines to their health. The risk is likely related to the perception of the vaccination event as uncontrollable and unknown. Some of them, through emotion-based intuitive reactions to danger, expressed a general concern that the vaccine could cause long-term health problems.

“*I am worried about how the vaccine will affect my health in the long run*” (#385)

Women have repeatedly expressed concern about their reproductive health and the possible impact of the vaccine on a fetus or breastfeeding infant. Women also argued the decision to postpone the vaccination due to the recommendation of the family doctor and emphasized their willingness to do it later.

“*I am pregnant and I am a bit fearful about the effects of the vaccine on the baby. I would like to be vaccinated after giving birth*” (#156);

“*My family doctor does not recommend that I get the vaccine while I am breastfeeding, as soon as I finish or when it’s safe, I will get vaccinated*” (#1,317)

Some HCWs relied on their past negative experiences with vaccination and referred to the recommendations of specialists regarding their health. Therefore, personal vaccination experience is an essential factor and characterizes risk perception as a non-intuitive and logical way to make a decision.

“*History of very severe anaphylactic reactions. Even manufacturers recommend not vaccinating. In addition, severe reactions to the flu vaccine*” (#561)

It is understood that some HCWs decided not to be vaccinated or to postpone it, for the time being, based on certain cases, on the advice of experts and their experience. Some of them, in an intuitive way of perceiving risk, expressed a general concern about negative consequences for their health and refuse to be vaccinated.

#### Theme: Risk perception toward COVID-19

The next significant factor influencing the decision to not get vaccinated is the low perception of the risk of contracting COVID-19 and not associating disease severity with risk for self.

“*I’m not part of a risk group*” (#1,243). “*I have never been vaccinated against anything and I am not going to. So far I have survived quite well*” (#1,000)

Some HCWs believed that the disease would be easy to treat and not dangerous even if they became infected, emphasizing young age as a risk-reducing factor and focusing on the body’s natural protective capabilities.

“*I don’t see the point, because it’s the same disease as the flu. We treat the flu with a family doctor and hospital, but if we get COVID, we stay at home and do nothing! People need good diagnosis and treatment instead of doing nothing and just getting vaccinated!*” (#1,104)

“*I have already been ill and do not see anything terrible in it. I am young*” (#952)

“*Let the body fight for itself. Let’s not disrupt the immune system with all kinds of vaccines*” (#493)

Some participants expressed the belief that it is enough to follow safety precautions.

“*I’m not in a risk group, I wear an ffp3 respirator everywhere, I don’t go anywhere, I order everything online, I don’t think I endanger myself or others*” (#171)

This theme also includes responses where HCWs indicated that they had recently contracted COVID-19 and were confident that they were immune to re-infection and that vaccination after the illness was not recommended. They mentioned the presence of COVID-19 antibodies after infection. In addition, some respondents stated that they were monitoring the level of antibodies after the illness or were sure that they had antibodies.

“*I got sick and I developed immunity. I don’t see the point in the vaccine*” (#1,368)

“*I was infected and have antibodies that I test regularly in the laboratory (with an upward trend)*” (#383)

#### Theme: Misinformation about COVID-19 vaccines

The lack of reliable information about COVID-19 vaccines was mentioned among HCWs. This theme includes responses from participants who stated a lack of reliable data and conflicting information. Some have expressed feeling overwhelmed and confused, which relates to a lot of negative information and a lack of long-term studies (as a source of reliable information).

“*Contradictory and incomplete information*” (#611)

“*A lot of negative reviews, and articles about complications and deaths. As well as the lack of information about the composition of vaccines and clinical trials. The exact side effects are unknown*” (#1,422)

Healthcare workers are expected to use scientific literature to obtain information. In the case of COVID-19 infection, information about the possible adverse effects of vaccines was actively discussed in the press and on social networks, medical professionals could likely be affected by ambiguous information, which led to doubts and mistrust.

#### Theme: Belief that vaccination is being imposed

The belief that the vaccine was also being imposed was mentioned among HCWs in some instances. This theme revealed the problem that vaccination is not voluntary and is being imposed by the workplace, which may promote vaccination hesitancy for some HCWs.

“*No argument for doing it. I don’t like violent imposition*” (#816)

#### Theme: Belief in conspiracy theories surrounding COVID-19

There were isolated cases of HCWs expressing their beliefs in conspiracy theories surrounding COVID-19. Some of the HCWs believed that the vaccination process is business-oriented and doubted the existence of COVID-19 and vaccination necessity in general.

“*Because I believe there is no such COVID-19*” (#1,116)

“*Business of pharmaceutical companies*” (#24)

### Coronavirus disease 2019 vaccination motivators

#### Theme: Belief in the effectiveness of the vaccine

The most frequently mentioned vaccination motivator was related to belief in vaccine effectiveness. Although the potential risks of side effects were not denied, the overall efficacy and safety of the vaccine were high. Respondents acknowledged that vaccination side effect risks are much lower than the disease-related risks. Furthermore, regarding COVID-19 vaccine knowledge, vaccination allows one to completely avoid infection or prevent severe illness, complications, and death.

“*We have two options, either get sick or get vaccinated. This infection is dangerous and unpredictable. I don’t want to play the Russian roulette*” (#473)

“*It protects me from serious illness and reduces the chance of getting sick*” (#109)

It is important to emphasize that respondents often stated that their motivation for vaccination is not only to protect themselves. Since HCWs work in high-risk settings, their concerns often revolve around saving relatives, friends, and other members of society, including promoting herd immunity by reducing the spread of the virus and its mutations.

“*Protecting parents and family*” (#626)

“*Vaccination is the most effective way to ensure the overall immunity of a large number of people (herd immunity). The only way to stop a mass disease*” (#451)

Most HCWs working in the medical system have obtained higher education and continue to improve their knowledge in seminars and courses that promote confidence and faith in medicine and science. As a result, confidence in efficacy is not only related to the COVID-19 vaccine but vaccination in general. Vaccination is perceived as an appropriate and successful means of resisting infectious diseases and, therefore, can give a sense of security.

“*Vaccination is one of the greatest medical advances and has already saved us from many infectious diseases!*” (#77)

Severe COVID-19 experienced by themselves or close acquaintances motivated respondents to get vaccinated and increased confidence in the efficacy of vaccines. Personal experience can give an impact on beliefs, and HCWs are a very specific group, who are not only particularly exposed to a high risk of infection but can also experience the most severe cases of illness in their workplace.

“*I don’t want to get sick because people I know were seriously ill and died*” (#662)

“*A year ago, I contracted this virus and got seriously ill, so I got vaccinated as soon as there was a chance*” (#432)

#### Theme: Benefits of easing COVID-19 restrictions

Due to the pandemic, severe restrictions were introduced, which negatively affected the professional and personal lives of all residents of Latvia. Therefore, removing or reducing these restrictions has become one of the main vaccination motivators among HCWs. A few respondents noted that they want the regulations to be lifted in general, while others emphasized specific restrictions in the workplace and the field of education, cultural life, or travel opportunities.

“*I don’t want to get tested twice a week, which is necessary to ensure safe working conditions*” (#779)

“*Vaccination would allow a return to normal life, to meet, shop, travel, go to the cinema, concerts, theatres, etc.*” (#595)

#### Theme: Responsibility

Working in the healthcare sector could explain specific subjective norms and a sense of responsibility. Respondents mentioned that their motivation to vaccinate was related to workplace risks and safety, including protecting patients and colleagues.

“*I am a medical person who works with severely ill people daily. This is my responsibility to them. To be healthy and not endanger others*” (#800)

“*If I get sick, there is a risk that my colleagues will get sick and will not be able to work either because they will be sick or will have to isolate themselves, and their patients will also suffer*” (#533)

The HCWs’ responsibility to the stability of the healthcare system promotes their motivation for vaccination, to reduce the workload of hospitals and laboratories. They believe that by getting vaccinated, the risk of hospitalization decreases. In addition, testing will be required less often.

“*To improve the epidemiological situation in the country and reduce the congestion of the health system*” (#1,308)

For HCWs, the role they play can be significant, as they reduce the doubts of others by their example. Therefore, some of the motivation to be vaccinated was increased by the example of colleagues. Others stated that they wanted to set a positive example for others by getting vaccinated.

“*I didn’t analyze much at the time. Everyone got vaccinated and so did I*” (#385)

“*Be an example to your loved ones, colleagues, friends*” (#1,185)

#### Theme: Restriction or pressure in case of non-vaccination

The healthcare system had some of the strictest epidemiological safety measures. Therefore, the possible existing work restrictions or other negative consequences in the case of non-vaccination were one of the motivating factors among HCWs. Although at the time data were collected, vaccination was not mandatory, respondents stated that they felt informal pressure.

“*If you have not been vaccinated, there is a lot of public pressure, you have to constantly fill in various surveys and listen to lectures*” (#627)

## Discussion

This study aimed to explore motivators and barriers to the COVID-19 vaccination of HCWs in Latvia. We found that the important themes of the barriers to COVID-19 vaccination among HCWs in Latvia were concerns about the effectiveness and safety of vaccines, perceived health risks of vaccination, risk perception toward COVID-19, misinformation about COVID-19 vaccines, belief that vaccination is being imposed, and belief in the conspiracy theories surrounding COVID-19. In addition to barriers, we identified significant motivators of HCWs to get vaccinated: belief in the effectiveness of the vaccine, benefits of easing COVID-19 restrictions, responsibility, and, in some cases, restrictions or pressure in case of non-vaccination.

Considering that the main goal of the COVID-19 vaccination strategy was to ensure the continuous functioning of the healthcare system and reduce the burden of mortality and morbidity, the organization and implementation of the COVID-19 vaccination campaign among HCWs were one of the priority tasks (to vaccinate the first high-risk group of the population in Latvia). It is stated that HCWs have a significant role in successful COVID-19 vaccination uptake and it was expected that they would administer, recommend and accept COVID-19 vaccines (World Health Organization, 2021). Previous studies have found that acceptance and intention to get vaccinated among HCWs are relatively high in most cases (Li et al., 2021; Lielšvāgere-Endele et al., 2021), although their arguments are not unambiguous.

It is known from other studies that attitudes toward the COVID-19 vaccine are strongly associated with vaccination intention. A negative attitude toward the vaccine significantly predicts COVID-19 vaccine refusal (Pogue et al., 2020; Guidry et al., 2021). Various quantitative studies reveal that the main vaccination barriers are related to concerns about safety and efficacy (Pogue et al., 2020; Biswas et al., 2021; Li et al., 2021; Woodhead et al., 2021), the insufficient number of clinical trials (Pogue et al., 2020; Fares et al., 2021), and the rapid vaccine development/approval process (Li et al., 2021). These findings support our study results, where the respondents’ viewpoints help reveal more detailed doubts, personal beliefs, and risk perceptions of COVID-19 and vaccination. We can note that HCWs’ concerns about the effectiveness and safety of vaccines arise out of distrust of the vaccine production and technology and dissatisfaction with the lack of clinical trials, which makes them feel like “test bunnies.”

Concerns about effectiveness and safety are closely related to perceived health risks which also exist among HCWs. We know from previous studies that potential vaccination side effects (Pogue et al., 2020; Biswas et al., 2021; Fares et al., 2021; Li et al., 2021) are a significant obstacle to an accepting attitude. Perceived potential health problems from the vaccines are often unknown long-term health issues and risks to reproductive health. It is a sensitive and uncontrollable topic related to emotion-based reactions. Although expressed barriers very often can be considered intuitive, in cases where there is proof of immunity to re-infection or a real (confirmed by previous experience) threat of anaphylactic reaction, vaccination refusal appears to be a rational and logical decision.

We can see that the risks of vaccination and COVID-19 are being considered. A low-risk perception (Li et al., 2021) for COVID-19 infection may be one of the hindering factors to vaccination. Risk perception covers beliefs that disease severity is not a risk to self, it is treatable, especially in those at a young age. Among some HCWs, it is expected that natural immunization and following safety precautions can be effective enough.

Much less common, but still relevant among Latvian HCWs, is the threat of misinformation. We find out that confusion and doubt can be provided by the lack of reliable data and conflicting information. Misinformation about COVID-19 was mostly spread on social media, and even HCWs are not protected from it (Datta et al., 2020; Aci et al., 2021; Woodhead et al., 2021). HCWs in India accepted that 68% of them have received inaccurate information from social media, family, and friends. Sometimes it is difficult to differentiate correct from incorrect information (Datta et al., 2020).

Rarely, but in some cases, HCWs expressed concerns that vaccination is imposed and stated that it creates negative emotions, resistance, and refusal. A sense of vaccination pressure among HCWs is related to institutional pressures and the possibility of dismissal (Aci et al., 2021). As a result, HCWs can decide not to voice their vaccine-related concerns, making their concerns more difficult to address (Heyerdahl et al., 2022).

Similar to the pressure theme, conspiracy-related statements during interviews appeared only a few times. We know from previous studies that vaccine hesitancy is linked to embracing vaccine conspiracy beliefs (Al-Sanafi and Sallam, 2021). In some cases, HCWs expressed concern that vaccination was driven by business interests rather than public health or that COVID-19 did not exist. Conspiracy-related concerns and belief in conspiracy theories can be related to distress, anxiety, low job, and life satisfaction of HCWs (Chen et al., 2020).

One of the key strategies of the World Health Organization (2021) is to create a positive social norm by highlighting those who get vaccinated. Therefore, it is crucial to understand the aspects of the HCW’s motivation. Previous evidence exists stating that vaccine acceptance and motivation to be vaccinated are related to trust in the safety and effectiveness of the vaccine (Fares et al., 2021; Li et al., 2021). Our findings highlighted this evidence with a deeper understanding of the HCW’s confidence in vaccine effectiveness. It is a most frequently mentioned theme in our study and covers the HCW’s general trust in vaccines. They believe that vaccines are the most effective way to avoid infection and can prevent complications. Vaccines are perceived not only as a personal benefit but also as a way to protect others and boost herd immunity. The results of the study show that the effectiveness and safety of vaccines are perceived differently among healthcare professionals, and this may be an argument for both acceptance and refusal.

Lifting or easing restrictions imposed to limit the spread of COVID-19 is another HCW argument for being vaccinated (Woodhead et al., 2021). HCWs see the benefit of vaccination and the willingness to lift all kinds of restrictions related to work and private life regulations.

Specific subjective norms, like the desire to promote and discuss vaccination (Manby et al., 2021) and willingness to protect patients (Manning et al., 2021), as well as high exposure to infection (Biswas et al., 2021; Li et al., 2021), are the significant motivators for vaccination among HCWs. Our study results reveal that HCWs’ responsibility was related to protecting patients and colleagues and the desire to decrease the healthcare system workload and be examples for others.

It is important to note that we identified work restrictions and pressure as a motivator to get the vaccine in some cases. At the same time, imposed vaccination was a hindering factor for others, pointing to the ambiguity and the dilemma that HCWs can face due to pressure. There is evidence that greater success and increased motivation can occur if the perceived pressure or influence is classified more as proactive encouragement (Woodhead et al., 2021).

This study suggests that HCWs in Latvia have different motivating factors and barriers that could influence or discourage their decision to receive the vaccine. It is worth indicating that the study results are more applicable to women, as they made up the majority of respondents. As there are significant differences between women’s and men’s willingness to be vaccinated (Zintel et al., 2022), men likely have different attitudes, motivators and barriers to COVID-19 vaccination than women. In context with our study respondents’ arguments, women more often have concerns about reproductive health and pregnancy. Knowledge gaps may exist in guidance on women’s health because there is proof that maternal and newborn health professionals rarely received training on COVID-19 from their health facility, and nearly all searched for information themselves (Semaan et al., 2020).

Nurses were also more inclined to express concerns in this study. Previous studies have found that nurses are more hesitant to vaccinate than other HCWs (Li et al., 2021). However, the rates of vaccination refusal are decreasing. Still, evidence-based strategies should be implemented to increase the uptake of COVID-19 vaccines among nurses to ensure their safety (Khubchandani et al., 2022).

Finally, it is essential to draw attention to the fact that of the 1,444 respondents in the main study, only 198 (13.7%) answered the question about vaccination barriers, while respondents were more willing to answer the question about vaccination motivating factors in 528 (36.5%) of the cases. The question of vaccination barriers against COVID-19 is likely a sensitive issue for HCWs. Therefore, they responded less to this question, and other research methods could be used to obtain a more accurate point of view.

## Implication

The results of our study have several implications. First, the study confirms the existence of vaccination barriers among Latvian HCWs and significant motivators. This qualitative approach explains what causes concern among HCWs regarding COVID-19 vaccines and what can increase motivation. Therefore, future campaigns could be focused on the specific vaccination barriers and vaccination motivators most prevalent among HCWs. Second, our findings can be used to focus on the groups of professionals most susceptible to vaccination hesitancy, female nurses. Third, this study’s qualitative results will help to create a better understanding of the survey’s quantitative results, which may contribute to a deeper understanding of the problems associated with barriers to vaccination among HCWs in Latvia.

## Limitations

There are some limitations to this study. First, online data collection does not allow for a clarification of the answers. Second, data used for this research were collected from March to May 2021, and it might not reflect the current state of affairs due to the COVID-19 situation, information about it and societal attitudes are subject to constant change. Third, at the time of the study, HCWs in Latvia started the vaccination process, but respondents were not divided into vaccinated and non-vaccinated groups within this study. Fourth, this study analyzed the part of the survey in which respondents in open-ended questions noted their motivators or barriers to vaccination, but the decision-making process was not considered. Fifth, the study included many more female participants than male participants, two groups that might have different experiences, perspectives, and awareness. Therefore, further studies should be performed separately to better understand the differences between vaccinated and vaccinated workers’ attitudes. Also, it is recommended that data analysis should be carried out in two gender-similar groups. To further improve understanding, it would be advisable to use other qualitative research methods, such as in-depth interviews and focus groups.

## Conclusion

The study found that among HCWs in Latvia, different motivating factors and barriers related to COVID-19 vaccination exist. A primary motivator for COVID-19 vaccination in HCWs in Latvia was the belief that vaccination is an effective method to prevent infection, severe illness, complications, and death. At the same time, the main barriers to COVID-19 vaccination were potential health risks and concerns about the effectiveness and safety of vaccines. As HCWs have a critical role in protecting themselves, the patients, and society, it is crucial to understand their arguments for and against vaccination to improve vaccination acceptance among HCWs by developing a targeted evidence-based information campaign about the effectiveness and safety of vaccines, with potential health threats, including women reproductive health, and COVID-19 infection risks. Furthermore, it is crucial to prevent conspiracy theories and alleviate informal pressure. This can be done by emphasizing the benefits to personal and public health that vaccination can provide and creating a positive social norm by citing other colleagues as examples.

## Data availability statement

The raw data supporting the conclusions of this article will be made available by the authors, without undue reservation.

## Ethics statement

Ethical review and approval was not required for the study on human participants in accordance with the local legislation and institutional requirements. Written informed consent from the patients/participants or patients/participants legal guardian/next of kin was not required to participate in this study in accordance with the national legislation and the institutional requirements.

## Author contributions

IM: conceptualization and funding acquisition. JK: methodology and supervision. SL-E, JK, and IM: validation. SL-E and EP: formal analysis. SL-E: investigation, resources, data curation, and writing—original draft preparation. ST: writing—review and editing. IM and JK: project administration. All authors have read and agreed to the published version of the manuscript.
